# Bioactive glass–ceramics containing fluorapatite, xonotlite, cuspidine and wollastonite form apatite faster than their corresponding glasses

**DOI:** 10.1038/s41598-024-54228-0

**Published:** 2024-02-18

**Authors:** Gloria Kirste, Altair Contreras Jaimes, Araceli de Pablos-Martín, Juliana Martins de Souza e Silva, Jonathan Massera, Robert G. Hill, Delia S. Brauer

**Affiliations:** 1 Otto Schott Institute of Materials Research, Friedrich Schiller University, Lessingstr. 12 (AWZ), 07743 Jena, Germany; 2 Fraunhofer Institute for Microstructure of Materials and Systems IMWS, Walter-Hülse-Str. 1, 06120 Halle, Germany; 3 Institute of Physics, Martin Luther University Halle-Wittenberg, Heinrich-Damerow-Str. 4, 06120 Halle, Germany; 4 Faculty of Medicine and Health Technology, Tampere University, Korkeakoulunkatu 3, 33720 Tampere, Finland; 5 Dental Physical Sciences, Barts and the London School of Medicine and Dentistry, Queen Mary University of London, Mile End Road, London, E1 4NS UK; 6 Leibniz Institute for Solid State and Materials Research (IFW), Helmholtzstr. 20, 01069 Dresden, Germany; 7 Present Address: Wilhelm Dyckerhoff Institut, Dyckerhoff GmbH, Dyckerhoffstraße 7, 65203 Wiesbaden, Germany

**Keywords:** Biomaterials, Biomedical materials, Glasses

## Abstract

Crystallisation of bioactive glasses has been claimed to negatively affect the ion release from bioactive glasses. Here, we compare ion release and mineralisation in Tris–HCl buffer solution for a series of glass–ceramics and their parent glasses in the system SiO_2_–CaO–P_2_O_5_–CaF_2_. Time-resolved X-ray diffraction analysis of glass–ceramic degradation, including quantification of crystal fractions by full pattern refinement, show that the glass–ceramics precipitated apatite faster than the corresponding glasses, in agreement with faster ion release from the glass–ceramics. Imaging by transmission electron microscopy and X-ray nano-computed tomography suggest that this accelerated degradation may be caused by the presence of nano-sized channels along the internal crystal/glassy matrix interfaces. In addition, the presence of crystalline fluorapatite in the glass–ceramics facilitated apatite nucleation and crystallisation during immersion. These results suggest that the popular view of bioactive glass crystallisation being a disadvantage for degradation, apatite formation and, subsequently, bioactivity may depend on the actual system study and, thus, has to be reconsidered.

## Introduction

Glass–ceramics (GC) derived from bioactive glasses are interesting materials for bone regeneration. Due to their remaining glassy matrix, they share some properties with bioactive glasses, one crucial feature being their capacity to degrade in aqueous solutions, causing the release of ions such as orthophosphate and Ca^2+^. As a result, apatite surface layer precipitation is promoted both in vitro and in vivo, with the apatite being biomimetic, i.e., chemically similar to the inorganic component of bone tissue^1^. This enables bonding to bone^2^ and, together with the degradation rate matching the rate of new endogenous bone tissue formation^3^, allows for implantation to fill bone cavities and support bone regeneration.

In some systems, the crystalline fraction of GC improves the mechanical properties compared to bioactive glasses, and, thus, serves to broaden the field of potential application^4–7^. However, generally crystallisation has been reported to cause slower degradation and reduced bioactivity compared to the corresponding glasses^5,6,8–12^. Nonetheless, the presence of a crystalline phase may even promote bioactivity, for example when pre-existing apatite crystals serve as nuclei for further apatite precipitation during immersion in physiological solutions^13,14^. Therefore, GC design needs to consider multiple aspects such as type of crystalline phases and microstructure, which are influenced by glass composition and heat treatment.

The GC in this study, obtained from bioactive glasses in the system SiO_2_–CaO–P_2_O_5_–CaF_2_, vary in their phosphate/silicate ratio (PSR) but have a constant silicate network connectivity (NC) of 2.11^15,16^, same as the well-known Bioglass 45S5. Fluoride addition to glasses allows for the crystallisation of fluorapatite (FAp) during heat treatment^17^ but also causes the pH rise upon immersion to be less pronounced^18^ and potentially allows for an increased bone density in vivo due to the incorporation of fluoride into the bone apatite^19,20^.

We chose alkali-free compositions to control the ion release behaviour. Established bioactive glasses with high sodium content degrade fast^21^, resulting in a burst of sodium ion release and, as a consequence, a pronounced pH rise and, subsequently, local cell toxicity^22,23^. In addition, the resulting fast degradation does not match the slower bone regeneration rate^24,25^.

The PSR in the glass has a pronounced influence on the apatite precipitation of soda lime silicate bioactive glasses during immersion. Given a constant NC, a larger concentration of phosphate in the glass promotes apatite precipitation while limiting the pH rise^13,26,27^. In addition, apatite crystallisation during heat treatment strongly depends on the phosphate content ^28,29^.

The aim of this study was to improve our understanding of the degradation behaviour of bioactive GC, particularly the effect of crystal phases, residual glassy matrix and their relative amounts. Analyses of solutions and samples after immersion by elemental quantification, powder X-ray diffraction and infrared spectroscopy (FTIR) were complemented by high-resolution imaging for micromorphological characterisation. Together, these results show that besides the relative amounts of crystalline and amorphous phases, nano-sized channels strongly affect GC degradation. This means that unlike for bioactive glasses, local microstructural variation is key and may cause the GC's degradation, ion release and apatite precipitation to even surpass those of the corresponding glasses.

## Results and discussion

### Composition of the residual glassy matrix and its effect on ion release in tris buffer solution

GC consist of crystalline phases as well as a residual glassy matrix, which both need to be considered when analysing the composition versus ion release relationship. In general, the amorphous matrix is more prone to degradation in aqueous solution than the crystalline phases. Hence, the residual glassy matrix in particular has to be considered when studying the degradation behaviour of GC during immersion. For the purpose of evaluating the residual glassy matrix’s composition, we compared the crystallised phases with the base glass. However, a quantitative assessment of the residual glassy matrix' composition was complicated by the fact that a direct calculation of the remaining amount of glassy phase was not possible by full pattern refinement of the XRD data. The reasons were the implementation of a simplified xonotlite model, which disregards its potentially present polytypism^30^ combined with non-systematically varying lattice parameters and pronounced lattice defects for cuspidine, xonotlite and apatite. Thus, the set of potentially relevant parameters for refinement was too extensive to allow for a reliable quantification of the amorphous background ^31^.

**Figure 1:**
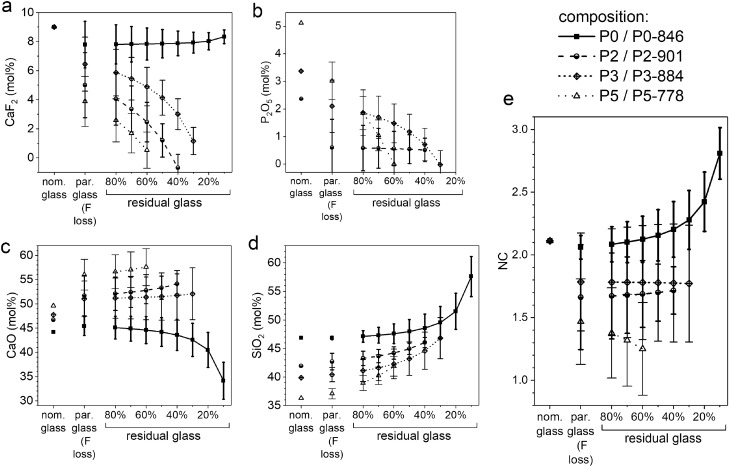
Changes in the composition of parent glasses (due to fluoride loss during melting^32^) and GC (due to incongruent crystallisation; concentrations estimated for different degrees of crystallinity; for calculation details see Supplementary File) compared to the nominal glass composition: (**a**) CaF_2_, (**b**) P_2_O_5_, (**c**) CaO and (**d**) SiO_2_ content; (**e**) network connectivity (NC; calculated using Eq. 1).

The volume percentages obtained from full pattern refinement of the XRD patterns are shown in Table 1. For the reasons stated above, amorphous fractions are not considered. All compositions contain an intermediate percentage of cuspidine (Ca_4_Si_2_O_7_F_2_; 18–52%) and varying amounts of the silicate minerals xonotlite (Ca_6_Si_6_O_17_F_2_) and wollastonite (CaSiO_3_). Additionally, an apatite phase was found, here assumed to be fluorapatite, FAp (Ca_5_(PO_4_)_3_F), or a fluoride-substituted apatite, as in previous immersion studies on fluoride-releasing bioactive glasses^18^. When comparing the crystalline phases with the initial glass compositions, we observe that the glasses crystallised incongruently (Fig. 1, Table 3). As expected, the apatite proportion increased with increasing PSR in the glass. Within the phosphate-containing compositions, the amount of xonotlite appeared to increase with apatite, whereas the proportion of wollastonite decreased. A minor amount of fluorite was detected in P0-846, i.e. the phosphate-free sample.

**Table 1:** Volume percentages of cuspidine, xonotlite, wollastonite and apatite present in the GC before immersion (not considering the residual glassy matrix), calculated by full pattern refinement.

Glass–ceramic	Cuspidine	Xonotlite	Wollastonite	Apatite
P0-846	24.0 ± 0.1	41.2 ± 0.2	34.3 ± 0.1	0.0 ± 0.2
P1-894	51.7 ± 0.2	0.3 ± 0.1	45.1 ± 0.2	0.1 ± 0.1
P2-901	49.3 ± 0.3	6.2 ± 0.1	41.3 ± 0.3	3.3 ± 0.1
P3-884	34.2 ± 0.1	24.2 ± 0.1	26.2 ± 0.1	15.4 ± 0.1
P4-816	25.6 ± 0.2	40.9 ± 0.2	3.6 ± 0.1	29.9 ± 0.2
P5-778	18.5 ± 0.2	42.8 ± 0.3	1.0 ± 0.1	37.7 ± 0.2

As shown previously, the glasses lost fluoride during melting^32^. Therefore, when comparing the nominal glass composition to the analysed parent glass composition (Fig. 1), results show an increase in CaO content and a decrease in CaF_2_ and P_2_O_5_ content from the nominal glass to the analysed parent glass composition. This was most pronounced for P5-778, i.e. the GC with the highest PSR^32^. The SiO_2_ content was not significantly affected by the underlying fluoride losses. As the actual relative amount of residual glassy matrix in the GC is not known, we estimated its composition and NC for different degrees of crystallinity (20–90%, Fig. 1). Details on the underlying calculations are given in the Supplementary File.

The decrease in CaF_2_ and P_2_O_5_ content by crystallisation of F- and P-containing phases allows for an estimate of the minimum amount of residual glassy matrix present in P2-901, P3-884 and P5-778. Due to the formation of the F-rich minerals cuspidine, xonotlite and FAp, the CaF_2_ content of the glassy matrix decreased upon crystallisation for most of the compositions (Fig. 1a). Since P0-846 was comparably low in cuspidine and cannot contain apatite (owing to the absence of phosphate), the CaF_2_ content was more or less independent of the degree of crystallinity. With the formation of apatite in the GC, the glassy matrix was depleted in P_2_O_5_ (Fig. 1b). This decrease in P_2_O_5_ was most pronounced for the sample with the highest PSR (P5-778), whereas for P2-901 the phosphate content appeared to be independent of the degree of crystallinity.

Estimating the degree of crystallinity for complete consumption of either CaF_2_ or P_2_O_5_ during formation of crystal phases suggests that the compositions contain rather large amounts of glassy matrix, which are at least 60% for P5-778, 30% for P3-884 and 40% for P2-901. Interestingly, both CaF_2_ and P_2_O_5_ appeared to be limiting factors for crystallisation in glasses P5 and P3.

Similar to bioactive glasses, the NC of the glassy matrix is a good indicator for the general tendency of a GC to degrade in water, provided that a sufficient amount of residual glassy matrix is present. We therefore expect trends in NC to be mirrored in the pH trends observed during immersion. Due to fluoride losses during melting, which have been shown to increase with P_2_O_5_ content in the glass^32^, NC decreased significantly for P_2_O_5_-rich composition P5-778 (Fig. 1e). A detailed description of how the fluoride loss reactions affect NC is given in the Supplementary File. For P3-884 and P2-901, a possible decrease in NC was difficult to observe as the experimental fluoride contents showed a relatively large variation^32^. As expected, the NC of P0-846 was least affected by fluoride loss. Interestingly, the change in NC caused by chemically incongruent crystallisation was comparably small for P2-901, P3-884 and P5-778.

Soda lime silicate and phospho-silicate glasses are well-known to cause a pH increase when immersed into aqueous solutions due to an ion exchange of network-modifying ions (in the present study Ca^2+^) linked to non-bridging oxygen units (NBO; ≡Si–O^−^) for dissociated H^+^ from the solution at the sample–solution interface^33^. This rise can be partially compensated for by buffering species such as phosphate ions^27^. A more detailed insight into the ion exchange process is obtained by compositional analysis of the solution, where the inorganic ions in solution are quantified.

**Figure 2:**
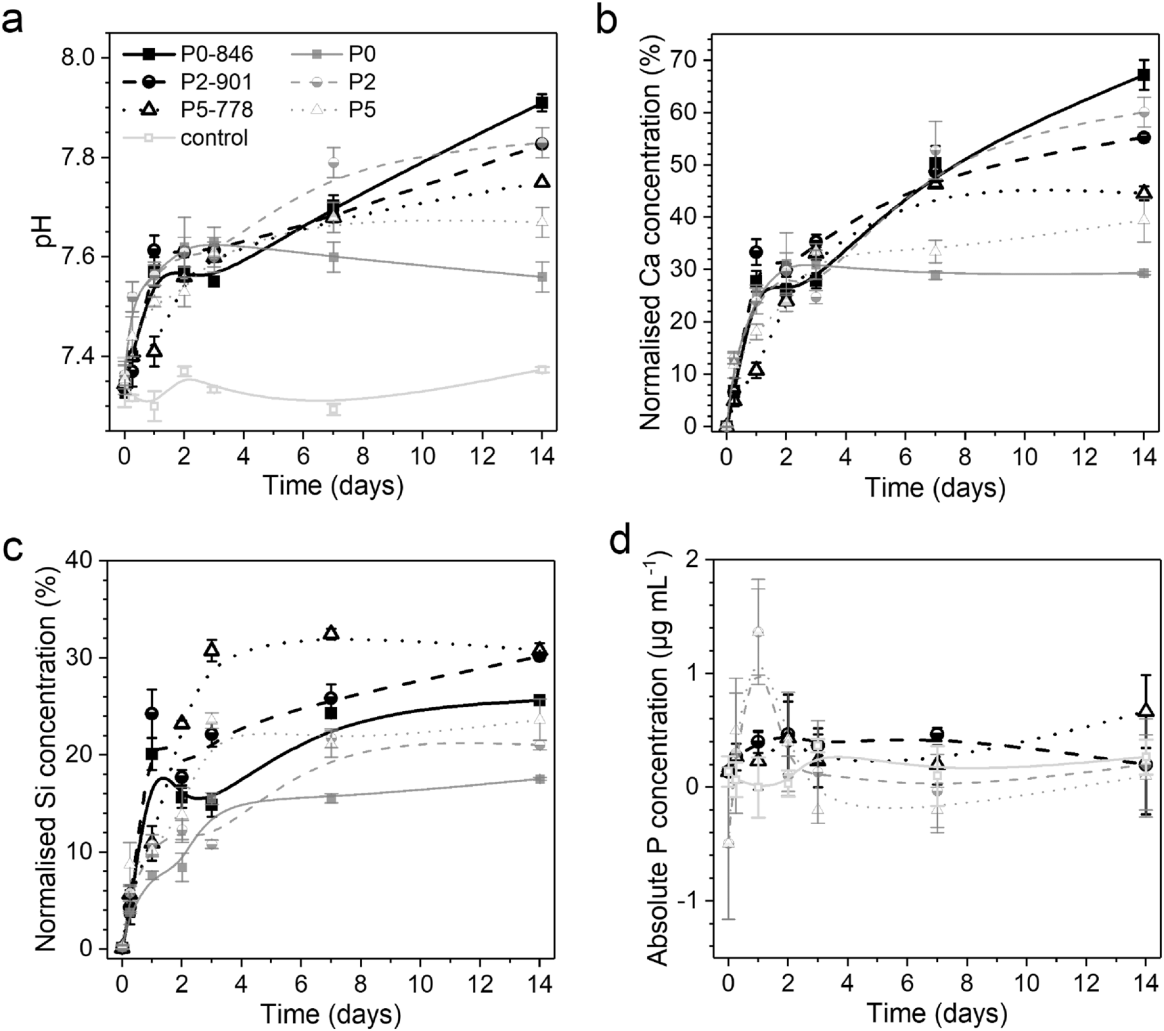
Characterisation of buffer solutions after immersion: (**a**) pH, relative (**b**) Ca and (**c**) Si ion concentrations and (**d**) absolute P concentrations of glasses and corresponding GC. (Ion concentrations in b and c have been normalised to the concentration of the respective ion in the untreated sample. Lines are drawn as visual guides only).

The pH rise (Fig. 2a) observed during early stages of immersion was less pronounced than that of conventional alkali ion-containing bioactive glasses^34^. Within the first 3 days of immersion the observed pH rise was similar for GC and their corresponding parent glasses (Fig. 2a), which indicates a large amount of residual glassy matrix with NC values comparable to the corresponding glass, as stated above. At 7 and 14 days of immersion, glass P0 gave the lowest pH value and P0-846 the highest, while the pH values for P2 and P2-901 or P5 and P5-778 were similar for glass and corresponding GC. The higher pH value for P0-846 compared to glass P0 is surprising, as one would expect the presence of crystalline phases (and, if applicable, the concomitant increase in NC of the residual glassy matrix) in P0-846 to result in an increased stability against water attack compared to P0. Instead, the opposite was observed, suggesting that, here, crystallisation reduced the chemical stability of the (phosphate-free) composition. A possible explanation may be that the interfacial boundaries between different crystal grains or crystal grains and glassy matrix act as selective sites for sample-solution reactions. Consequently, gaps forming at these intensified reaction sites facilitate water attack until finally fragmenting the immersed GC particles, see further below. In the phosphate-containing compositions, this effect was not observed, possibly because even prior to crystallisation distinct phase boundaries were already present, owing to the presence of pronounced phase-separation droplets in the parent glass as described in our previous study^15^. Hence, an equally strong sample/solution interaction at interfacial boundaries was enabled for both phosphate-containing glasses and GC.

As Ca^2+^ ions were the only network-modifying ions present, the trends in their relative concentrations in Tris buffer resembled the pH trends (Fig. 2b). Ca concentration curves of GC and corresponding glass were similar, matching our observations of opposite effects of crystallisation on CaO and CaF_2_ contents in the residual glassy matrix counterbalancing each other (Fig. 1a, c). At 14 days of immersion, the high-PSR samples P5-778 and P5 showed lower relative Ca concentrations compared to the low-PSR samples P2-901 and P2. A plausible explanation is that released Ca^2+^ ions were consumed during apatite precipitation, which was more pronounced for compositions with higher phosphate contents (higher PSR), as confirmed by full pattern refinement of XRD patterns of samples after immersion (Fig. 4). Thus, the GC behave similar to the corresponding glasses^15^.

Apatite precipitation in solution is limited by the amount of phosphate available. As Tris buffer is inherently phosphate-free, all phosphate needs to be released from the glasses or GC first. However, part of the phosphate present in the GC is already chemically bound as FAp and, thus, unlikely to be released at the near-neutral pH conditions used here^35^. As a consequence, the phosphate content in the residual glassy matrix is important for phosphate release and subsequent apatite precipitation. As shown in Fig. 1b, the glass with the largest initial PSR, P5, resulted in a GC with the lowest possible P_2_O_5_ content in the glassy matrix due to fluoride loss and crystallisation. Still, XRD showed that apatite did precipitate during immersion of all phosphate-containing GC including P5-778 (Fig. 4). This indicates that some phosphate release must have occurred and, therefore, suggests that the glassy matrix was not completely depleted in phosphate ions.

**Figure 3:**
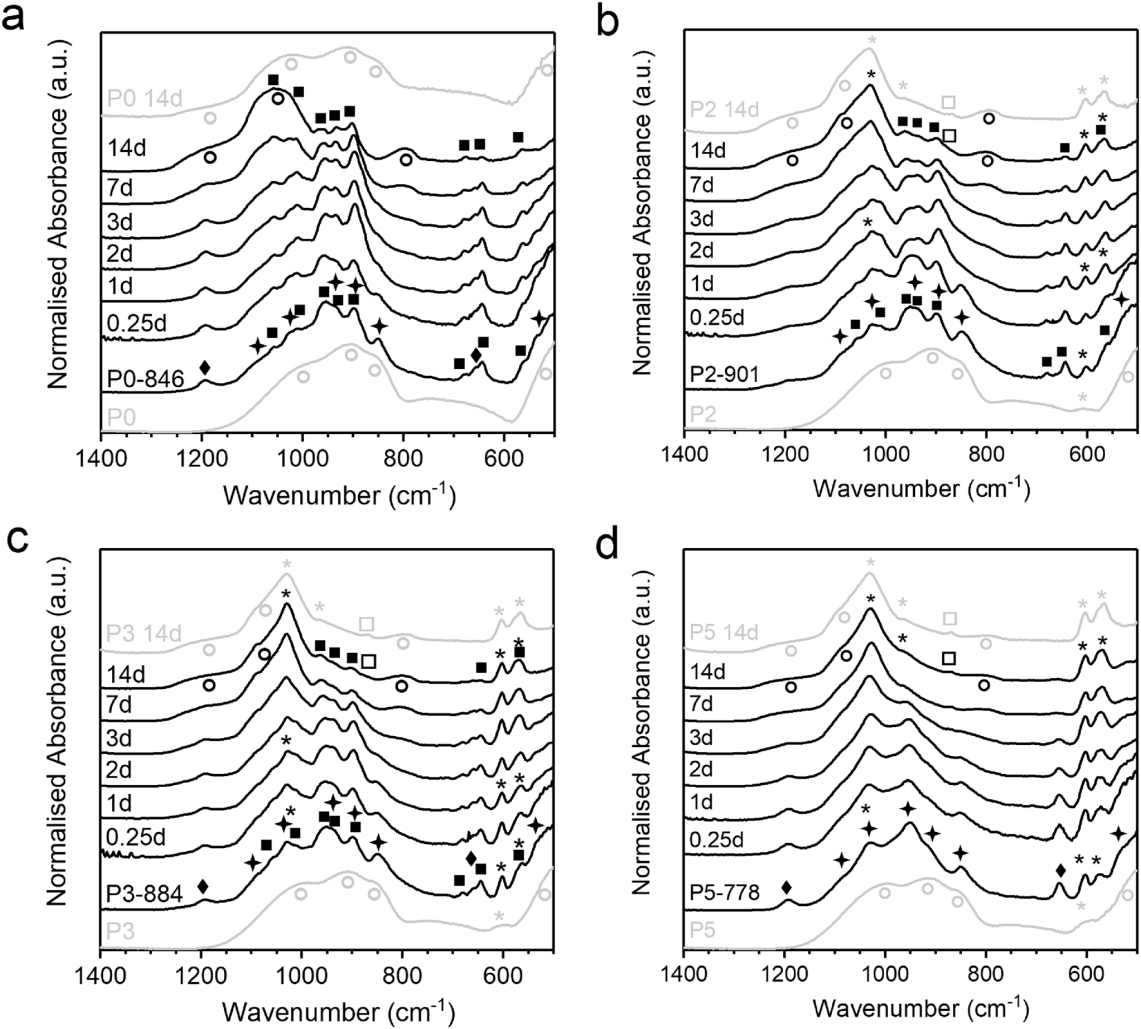
FTIR spectra at different time points of immersion for (**a**) P0 and P0-846, (**b**) P2 and P2-901, (**c**) P3 and P3-884, (**d**) P5 and P5-778. Parent glasses for comparison plotted in grey, labelling of vibration bands: (circle) amorphous Si–O, (star) cuspidine, (filled square) wollastonite, (diamond) xonotlite, (asterik) apatite/phosphate, (square) carbonate.

Ion concentrations in solution depend on the amounts of ions released from the sample during immersion as well as on the consumption of ions during precipitation of solid phases. Absolute P concentrations after immersion of GC were comparable to those of the phosphate-free control solution (Fig. 2d). For the glasses, P2 and P5 showed significant P concentrations at 1 day only. pH results (Fig. 2a) showed that ion release occurred from all samples. The difference between glass and GC therefore originated from different apatite nucleation conditions. For crystallisation of solid phases from aqueous solutions, nucleation often is the limiting step. The GC already contain apatite crystals from the heat treatment (Fig. 3), and these apatite crystals can act as nuclei for apatite precipitation from solution for P2-901 and P5-778. By contrast, during immersion of glasses, nuclei need to form first during immersion before apatite precipitation can occur, which was confirmed at 1 day of immersion ^15^.

When comparing the relative Si concentrations in solution, GC showed larger values than their corresponding parent glasses (Fig. 2c). In contrast to pH and Ca concentrations, this difference between glasses and GC was observed as early as at 1 or 2 days of immersion. This suggests that the increase in Si release in GC may be related to differences in initial release. Further trends in the evolution of the relative Si concentrations were challenging to interpret, since the solubility of Si species is affected by many factors, including pH and the presence of other species in solution. Moreover, the effects of material-related properties like internal interfaces, NC and phosphate content of the glassy matrix are difficult to take into consideration here due to the unknown degree of crystallinity in the GC.

### Structural and compositional characterisation

FTIR and XRD data show the relative amounts of initial and precipitated crystal phases changing with immersion time. At the same time, a change in the amorphous silicate structure was observed analysing the Si–O vibrational bands of the FTIR spectrum together with the amorphous halo in the XRD patterns (Suppl. Figure 1). The similarity between the NC of the GC's residual glassy matrix and that of the corresponding glasses (before immersion) was confirmed by a similarity in structure, illustrated in the FTIR spectra (Fig. 3). Here, the narrow bands of the crystalline species can be described as superimposed on the broader bands of the parent glass. The large amount of network modifiers in the residual glassy matrix caused strong signals at 920 and 860 cm^−1^, associated with the Si–O stretching mode in SiO_4_ tetrahedra with either one or two NBO^36^.

Upon immersion, the intensity of these NBO bands decreased because of Ca^2+^ ion leaching and NBO being replaced by silanol groups (Si–OH). Due to the formation of an ion-depleted silica-gel layer, a band at 790 cm^−1^ developed, which we assigned to Si–O–Si bond vibrations between two adjacent SiO_4_ tetrahedra in this ion-depleted silica-gel layer ^18^. Additionally, the bridging Si–O–Si bending band shifted from 1000 to 1070 cm^−1^ and its intensity increased. Interestingly, the Si–O–Si rocking mode band at 500 cm^−1,^^37^, which was very strong for both parent glasses and GC, retained its position and intensity for glass P0 only. Likewise, the bands at 920 and 860 cm^−1^ were still distinctly clear in P0 at 14 days of immersion. Together with pH and ion release behaviour, this indicates that P0 is not very reactive and that only the surface underwent ion exchange albeit without formation of an apatite layer (as no phosphate was present). By contrast, the corresponding GC (P0-846) showed more ion release with a pronounced change in glass structure due to silica gel formation. As mentioned above, this effect is likely to be connected to the GC’s large internal interface between crystal phases and glassy matrix.

Accordingly, the increased ion exchange in P0-846 is illustrated in the diffraction patterns of Suppl. Figure 1a. Here, the typical shift of the amorphous halo from approximately 30–22°2θ was observed for P0-846 but not for P0. Interestingly, the intensity of the GC’s amorphous halo was increasing with immersion time, which is in contrast to the changes observed for the corresponding glasses^15^. Especially for P0-846, P1-894 and P2-901 at 14 days of immersion a pronounced amorphous halo was observed, showing a remarkably larger intensity than the initial amorphous halo shown in the GC’s diffraction patterns before immersion. This finding contradicts the theory that formation of an ion-depleted silicate phase occurs solely from an amorphous glassy matrix. It is known from geochemical studies that an amorphous ion-depleted silica-gel layer can form at the interface of silicate minerals and aqueous solutions as well^38^. Hence, we consider it more likely that some of the GC's crystalline silicate phases are involved in exchanging Ca^2+^ for H^+^ ions and that, thus, those crystalline silicates participate in the formation of the ion-depleted silica-gel layer at the sample surface while reducing the overall silicate crystallinity of the sample. This hypothesis is supported by the close resemblance of the FTIR spectra of phosphate-containing GC and their respective parent glasses at 14 days of immersion (Fig. 3 and Suppl. Figure 2), suggesting a similar occurrence of ion-depleted silicate after immersion despite the differing amount of glassy matrix before immersion.

Additionally, the participation of crystalline silicates in the ion release agrees with the similarity of results for glasses and corresponding GC as shown by pH results and Ca^2+^ concentration in solution, since the reduced amount of glassy phase available in the GC would be expected to result in a less pronounced increase in both pH and Ca concentration. In the same context, the pronounced silica-gel layer formation for P0-846 agrees with ion release results: due to the pronounced condensation of silica in solution to form a silica gel, a maximum release of Ca is possible without a concomitant increase in Si release (Fig. 2b, c). The trend of decreasing intensity of the amorphous halo at 14 days of immersion with increasing PSR of the GC’ composition corresponds to an analogous trend for the immersed parent glasses, as expected for the higher SiO_2_ content in glasses with low PSR^15^.

With increasing PSR of the GC composition, the FTIR spectra of the immersed samples were increasingly dominated by phosphate-related features, i.e. the developing band at 1030 cm^−1^ and the characteristic split band between 570 and 600 cm^−1^ (Fig. 3b–d), reported as a P–O bend in crystalline apatite phases^10,39^. This agrees with progressing apatite precipitation as suggested by the Ca release data shown above, X-ray diffraction results (Suppl. Figure 1) and studies published in the literature^27^.

**Figure 4:**
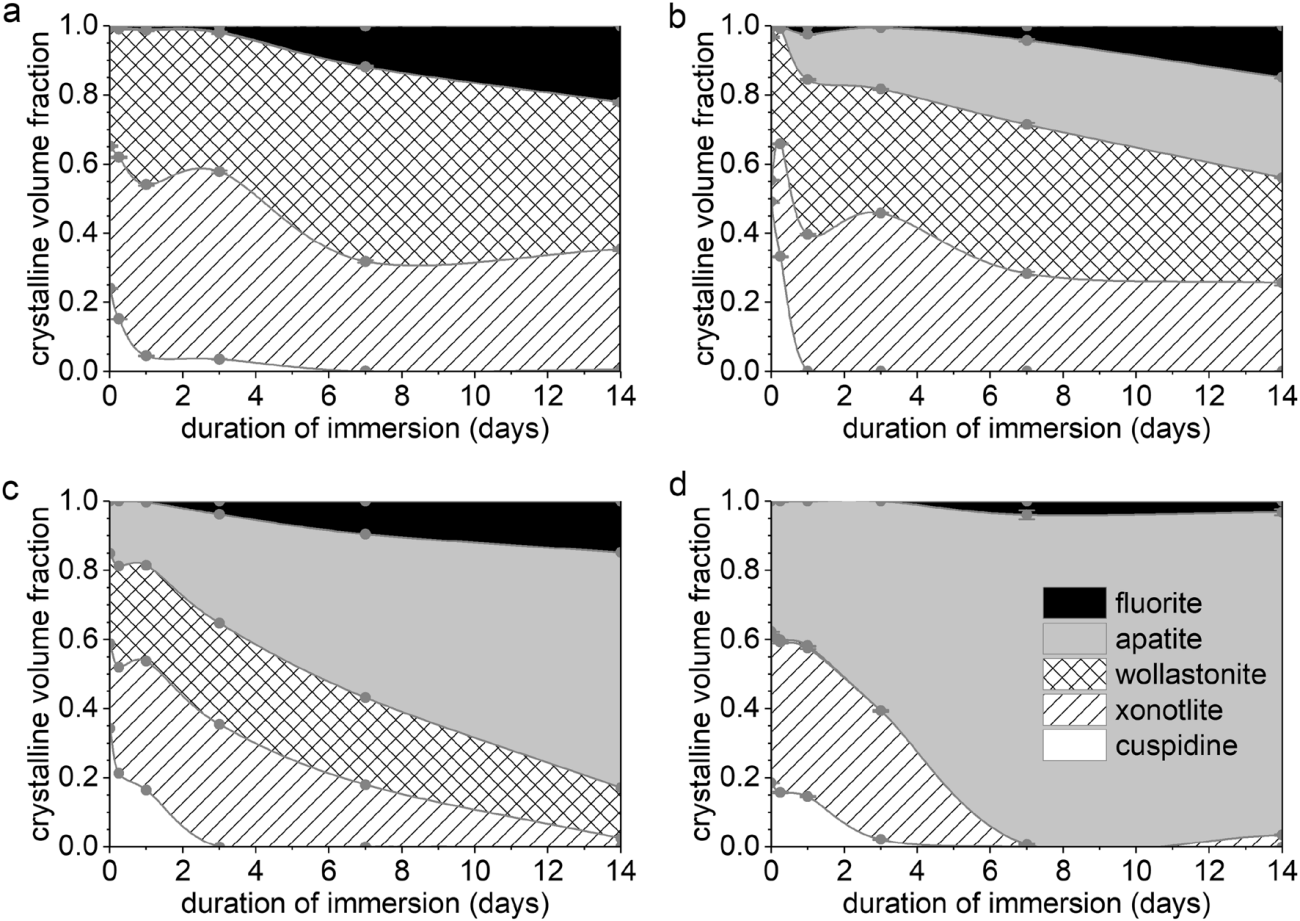
Volume fractions of cuspidine, xonotlite, wollastonite, apatite and fluorite within the crystalline share of the GC at different time points of immersion, calculated by full pattern refinement for (**a**) P0-846, (**b**) P2-901, (**c**) P3-884 and (**d**) P5-778. (Lines are drawn as a visual guide only, original diffraction patterns depicted in Suppl. Figure 1.)

From full pattern refinement of the XRD data, quantitative information was obtained on crystal phases present over immersion time (Fig. 4 and Suppl. Figure 3). Although the phosphate content of the residual glassy matrix may be considerably reduced, the increase in the relative amount of apatite during immersion appears to be correlated to the initial PSR of the GC. During 14 days of immersion the apatite fraction increased by about 13% for P1-894, 26% for P2-901 and 53% for P3-884 (Fig. 4a–c and Suppl. Figure 3). An even higher increase in PSR, however, did not seem to improve apatite precipitation further, as shown for P4-816 and P5-778 (58% and 56%, respectively). When interpreting these numbers, we need to consider that they were obtained from normalisation to the relative fractions of all crystalline components, and that silicate crystallinity decreased during immersion, thereby potentially affecting quantification. XRD patterns also reveal a larger crystallinity of the apatite precipitated on the immersed GC samples compared to the immersed glass samples, as indicated by the larger ratio of apatite reflection intensity to amorphous halo intensity, as well as the narrower shape of the apatite reflections in the GC (Suppl. Figure 1).

**Figure 5:**
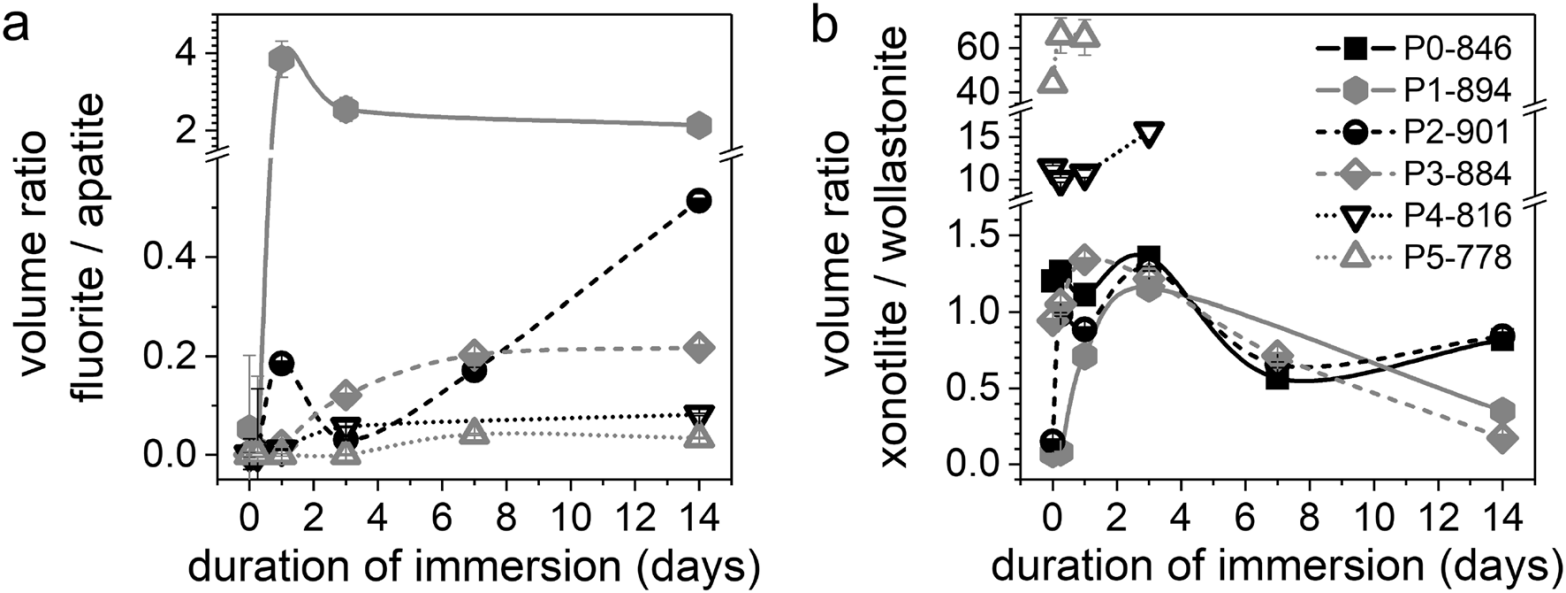
Evolution of volumetric ratios among crystalline phases in GC during immersion: (**a**) Fluorite-to-apatite and (**b**) xonotlite-to-wollastonite. (For samples P4-816 and P5-778, the xonotlite-to-wollastonite ratio at later time points could not be calculated because of very low wollastonite concentrations.)

Fluorite, CaF_2_, precipitated alongside apatite as an additional fluoride-containing mineral phase (Fig. 3) as observed by XRD. Co-precipitation of fluorite with apatite has previously been reported for low-phosphate F-containing bioactive glasses^13,34^. The presence of fluorite indicates an excess of Ca^2+^ ions and F^−^ ions in the solution that could not react towards apatite precipitation owing to a lack of available phosphate ions. Co-precipitation of apatite and fluorite was observed both in the GC and the parent glasses (Fig. 5, Suppl. Figure 1) ^15^.

The fluorite-to-apatite ratio after immersion decreased with increasing PSR in the composition (Fig. 5a). However, these changes in co-precipitation were not the same for all GC. For P5-778, both precipitated phases reached a stable volume fraction at 7 days, indicating that no further leaching from the GC and subsequent precipitation occurred (Fig. 4d). This agrees with the plateau observed for Ca and Si concentrations in solution during immersion of P5-778 (Fig. 2b,c). Even though P4-816 and P3-884 may not have reached stable volume fractions of apatite and fluorite at 7 days, they showed a stabilisation of the fluorite-to-apatite ratio, similar to P5-778. By contrast, P1-894 and P2-901 showed a drop in the fluorite-to-apatite ratio between 1 and 3 days of immersion. Afterwards, the fluorite-to-apatite ratio appeared to stabilise for P1-894, whereas a continuous increase was observed for P2-901. These differences in behaviour cannot be explained by the degradation behaviour of the residual glassy matrix alone but are probably affected by the crystalline phases and their distribution within the GC samples as well.

Even though crystalline silicates typically show a better stability in aqueous solutions than comparable amorphous phases, considerable leaching of alkali or alkaline earth ions and solubility of calcium silicate minerals is well-known from slags and cements^40,41^. Hence, the decrease in the GC’s silicate crystallinity, caused by ion exchange in the silicate minerals, is accompanied by the degradation of the crystalline species. As shown in Fig. 4, the amounts of different crystalline silicate phases did not decrease at the same rate, i.e. maintaining their ratio, as expected for a simple process of newly formed precipitates exceeding the share of initially existing silicates. Instead, the silicate minerals degraded in a certain sequence observed for all GC:

The first mineral to degrade was cuspidine. For samples with low PSR (P_2_O_5_ content < 3 mol%) this process was especially noticeable, leaving an amount of less than 25% of the initial cuspidine concentration at 1 day of immersion (Fig. 4a, b). Cuspidine concentrations decreased more slowly for samples with a higher PSR (P_2_O_5_ content > 3 mol%), which may be related to the larger absolute Ca^2+^ ion concentration in the solution (Fig. 1b). However, a relationship between cuspidine stability and F^-^ or PO_4_^3−^ ion concentration in solution cannot be excluded. The decrease in cuspidine concentration can also be correlated with the FTIR spectra (Fig. 3). Although Si–O bands of wollastonite and cuspidine overlap, the characteristic cuspidine band at 850 cm^−1^ is a good indicator for the presence of cuspidine^42^. Hence, a decrease in this band’s intensity strongly indicates a decrease in cuspidine content, in good agreement with the diffraction data.

Such degradation of cuspidine is plausible as it is known to release relatively large amounts of F^−^ and Ca^2+^ ions in aqueous solutions at pH 7–8 and therefore is less stable than fluorite or FAp^43,44^. The faster degradation of cuspidine compared to the other silicate phases present is probably related to its weakly polymerised silicate structure^40^ (disrupted sorosilicate with very weak [Si_2_O_7_]^6−^-dimer band at 715 cm^−1^, Fig. 3)^42,45^. On the other hand, it may also be related to the GC’s microstructure. As we have previously shown in a study on the GC before immersion^29^, the crystalline silicates in the present GC are intergrown on a sub-micron length scale due to simultaneous crystallisation. Elemental mappings allowed for interpreting the resulting microstructure as cuspidine and xonotlite being particularly intergrown with each other whereas wollastonite crystals were separate. Accordingly, the tendency to selectively dissolve cuspidine and xonotlite is supposedly increased as a result of their larger surface area, as well as other structural defects occurring from the simultaneous precipitation.

In samples with low PSR (P_2_O_5_ content < 3 mol%) the pronounced cuspidine degradation at 1 day of immersion occurred simultaneously with a temporary peak in Si ion concentrations (Fig. 2c, samples P0-846, P2-901). This finding indicates that the reaction of cuspidine with the solution was not merely limited to an ion exchange of Ca^2+^ for H^+^. Instead, a less selective dissolution of the silicate mineral was observed, including disintegration of Si–O–Si bonds, which is characteristic for silicate crystals of a poorly connected structure^38^. Equally, the fluorite-to-apatite ratio showed a short-time maximum at 1 day of immersion (Fig. 5a, samples P1-894, P2-901). Since the formation of fluorite is associated with large fluoride concentrations in solution, this also points at cuspidine dissolution remarkably affecting the ion concentrations in solution.

However, the effects of distinct cuspidine degradation in samples with low PSR did not lead to a lasting boost of relative Si concentration or fluorite-to-apatite ratio. Instead, both values decreased again from 1 day of immersion, indicating that part of the Si and F release from cuspidine was compensated for by incorporation into a newly formed solid phase. Regarding the decrease in released Si, a condensation of amorphous silica or the growth of another crystalline silicate phase can be assumed. For fluoride, the latter is more probable, pointing at an increase in the amount of xonotlite, since wollastonite is not known to incorporate fluoride. Confirmation is provided by the increasing trend of the xonotlite-to-wollastonite ratio within the first 3 days of immersion for all samples (Fig. 5b). Here, the xonotlite gain was especially pronounced for samples P1-894 and P2-901, which also showed the most pronounced degradation of cuspidine.

Following a brief initial increase in concentration, xonotlite was the next silicate mineral to degrade. In the FTIR spectra (Fig. 3), the decrease in xonotlite is shown by the weakened intensity of the characteristic bands at 1190 and 660 cm^−1,^^46,47^. Simultaneously, a decrease in xonotlite volume fraction (Fig. 4) and xonotlite-to-wollastonite ratio (Fig. 5b) was observed. In comparison with the degradation of cuspidine, the decrease in xonotlite concentrations occurred more gradually and does not reach depletion within 14 days of immersion.

Nevertheless, the pronounced decrease in xonotlite content for the GC with high PSR is surprising, since it is the most polymerised silicate crystal phase in this study, i.e. the silicate mineral with the highest NC. Based on the silicate NC, one would expect cuspidine (with its Si_2_O_7_^6−^ pyrosilicate anions, possibly disrupted into SiO_4_^4−^ orthosilicate anions, NC ≤ 1) to show more pronounced Ca release than the chain silicate wollastonite (SiO_3_^2−^ metasilicate anions, NC = 2)^40^. followed by the double-chain silicate xonotlite (Si_6_O_17_^10−^ anions, NC = 2.33)^30^. Here, xonotlite stability may be reduced, however, because of its intergrown microstructure with cuspidine. Especially once cuspidine degradation has started to progress, xonotlite degradation may be facilitated due to interfacial reactions with the immersion medium.

Interestingly, the most pronounced decrease in xonotlite concentrations between 3 and 7 days of immersion coincided with a large increase in fluorite concentration for all samples (Fig. 4). This may indicate xonotlite's role in delaying fluorite precipitation, most likely by temporarily binding fluoride from the dissolving cuspidine, even though stoichiometric xonotlite contains less fluoride than cuspidine. We further assume FAp formation to occur at the expense of xonotlite, xonotlite’s good performance at absorbing phosphate and its use as a seed crystal for apatite formation has been described by Chen et al^48^. Our assumption was further supported by the observation that the low-PSR samples retained a larger fraction of xonotlite at 14 days of immersion, since there was not enough phosphate available in the Tris buffer solution to allow for a substantial transformation of xonotlite into FAp. Xonotlite’s phosphate-binding properties may also be the reason why distinct additional FAp precipitation was observed for high-PSR GC despite the reduced P_2_O_5_ content in their residual glassy matrix (Fig. 1b).

Hardly any Si release was observed during xonotlite degradation in sample P5-778 (Fig. 2c, Fig. 4d), which indicates that the degradation process of xonotlite was limited to ion-release of Ca^2+^ and F^−^ combined with ion exchange of Ca^2+^ for H^+^. Even though xonotlite has been reported to dissolve congruently when immersed in water^41^, we did not observe disintegration of joined SiO_4_^4−^ tetrahedra upon xonotlite degradation. Nevertheless, the leached xonotlite reorganised its SiO_4_^4−^ tetrahedra in a structure similar to ion-depleted silica-gel, as indicated by the temporary correlation between the decreasing intensity of xonotlites bands (at 1190 and 660 cm^−1^) and the increasing intensity of the broad amorphous Si–O bands (at 1200, 1070 and 790 cm^−1^) in FTIR spectra (Fig. 3).

In our study, wollastonite appears to be the silicate crystal phase with the lowest degradation tendency, as its relative volume fraction did not decrease noticeably during immersion in Tris buffer solution (Fig. 4). As in P5-778 the initial wollastonite content was very small, however, its detection after immersion was impeded by the substantially increased apatite concentration. In the FTIR spectra of P0-846, P2-901 and P3-884, wollastonite’s characteristic bands between 825 and 975 cm^−1^ were present but their intensity was decreased due to superposition by an amorphous Si–O and a phosphate band in this wavenumber range. It is interesting to note that the trend of initially high wollastonite content for low-PSR GC^29^ transformed into a large fraction of residual silicate during immersion, whereas high-PSR GC were drastically depleted in silicate at 14 days of immersion.

### Microstructure after immersion

**Figure 6:**
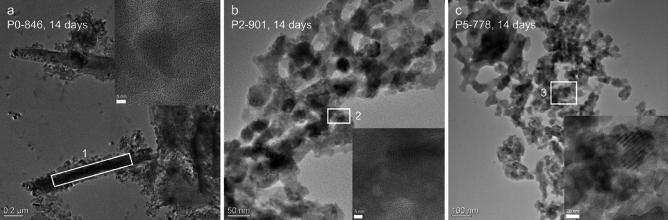
TEM micrographs at different magnifications of the GC powders of (**a**) P0-846, (**b**) P2-901 and (**c**) P5-778 after 14 days immersed in Tris buffer. Insets display details of crystalline areas, showing the crystalline planes.

TEM images of powder P0-846 at 14 days of immersion are displayed in Fig. 6a, showing stacked powder particles as well as their micro- and nanostructure. At the edges of some particles, elongated structures of several hundreds of nanometres in length were observed. The features highlighted by white rectangles seem to be formed by two of these elongated structures. EDX elemental quantification in this area (Table 2) showed a Ca:Si atomic ratio of nearly 1:1. This matches xonotlite and wollastonite, which according to XRD were present in equal amounts at 14 days of immersion (Fig. 4a). This observation agrees with the blade-like or needle-like morphology reported for both minerals in the literature^47,49,50^. However, the relatively large amount of fluoride detected in this area suggests xonotlite or a combination of wollastonite and fluorite. In addition to the elongated structures, crystalline planes were observed at higher magnification in some areas (inset in Fig. 6a).

**Table 2:** Atomic concentration (at%) of the selected areas in Fig. 6. The analytical accuracy of the EDXS measurement is around 2%.

Position	Si	Ca	F	P	O
P0-846 (Fig. 6a)	23.5	25.0	5.0	–	46.5
P2-901 (inset Fig. 6b)	14.2	15.8	–	8.2	61.7
P5-778 (inset Fig. 6c)	12.9	19.8	1	6.6	59.7

Powder particles P2-901 at 14 days of immersion are displayed in Fig. 6b. Their morphology was different from those in P0-846, showing channel-like structures with open spaces of several tens of nanometres in diameter within the particles. In one of our previous studies, we showed that theses pores or voids were not present in the bulk GC before immersion^29^. Hence, their presence may be related to the degradation of residual glassy matrix or of cuspidine, the latter having been present in large quantities in P2-901 before immersion. Since the ion release from residual glassy matrix and cuspidine was especially pronounced during the first few days of immersion, it is assumed that this perforated structure started developing early on. According to XRD results (Fig. 4b), the crystalline areas observed here (inset in Fig. 6b) must be related to xonotlite, wollastonite, apatite or fluorite. EDX analysis performed in one of the crystalline regions (inset of Fig. 6b) shows a Ca:Si ratio of nearly 1:1 (Table 2), suggesting the presence of wollastonite or xonotlite. However, the Si content was comparably low, the P content relatively high and no F was detected. Hence, we assume that two crystal phases, wollastonite and apatite, contributed to this result.

In addition to P0-846 and P2-901, a sample of P5-778 at 14 days of immersion was investigated by TEM. The micrograph in Fig. 6c shows a morphology similar to that of P2-901, but presenting an even more open structure. Larger voids emerged due to the increase of silicate network dissolution with increasing PSR (Fig. 4 and Suppl. Figure 1). The branching of the powder particles may be interpreted as a formation of the typical needle-like FAp crystals. EDX elemental quantification in the crystalline area depicted in the inset of Fig. 6c shows the Si content to be lowest in the immersed P5-778 particles (as expected from ion release and XRD data). Interestingly, the Ca:Si ratio of 1.5 reflects the initial Ca:Si ratio of the untreated P5 glass, despite XRD (Fig. 4d) suggesting hardly any crystalline silicate being left after immersion. Considering the relatively large P content, this indicates the presence of FAp together with some ion-depleted residual glassy matrix or leached xonotlite in the probed area.

TEM results suggest that GC degraded through formation of nano-scale channels by removing residual glassy matrix and cuspidine during immersion in aqueous solution. As stated above, an increase in PSR seems to result in an increased amount of residual glassy matrix (Figs. 1a,b, 5). In addition, orthophosphate clusters in the residual glassy matrix induced a higher solubility of the amorphous silicate network (Fig. 2c), and, as a result, the phenomenon of channel formation during immersion was more pronounced with increasing PSR of the GC. By contrast, phosphate-free P0-846 with its more inert residual glassy matrix did not show any channel formation. Its increased Si and Ca release (compared to parent glass P0, Fig. 4b, c) solely originated from favoured leaching at phase interfaces and cuspidine dissolution.

**Figure 7:**
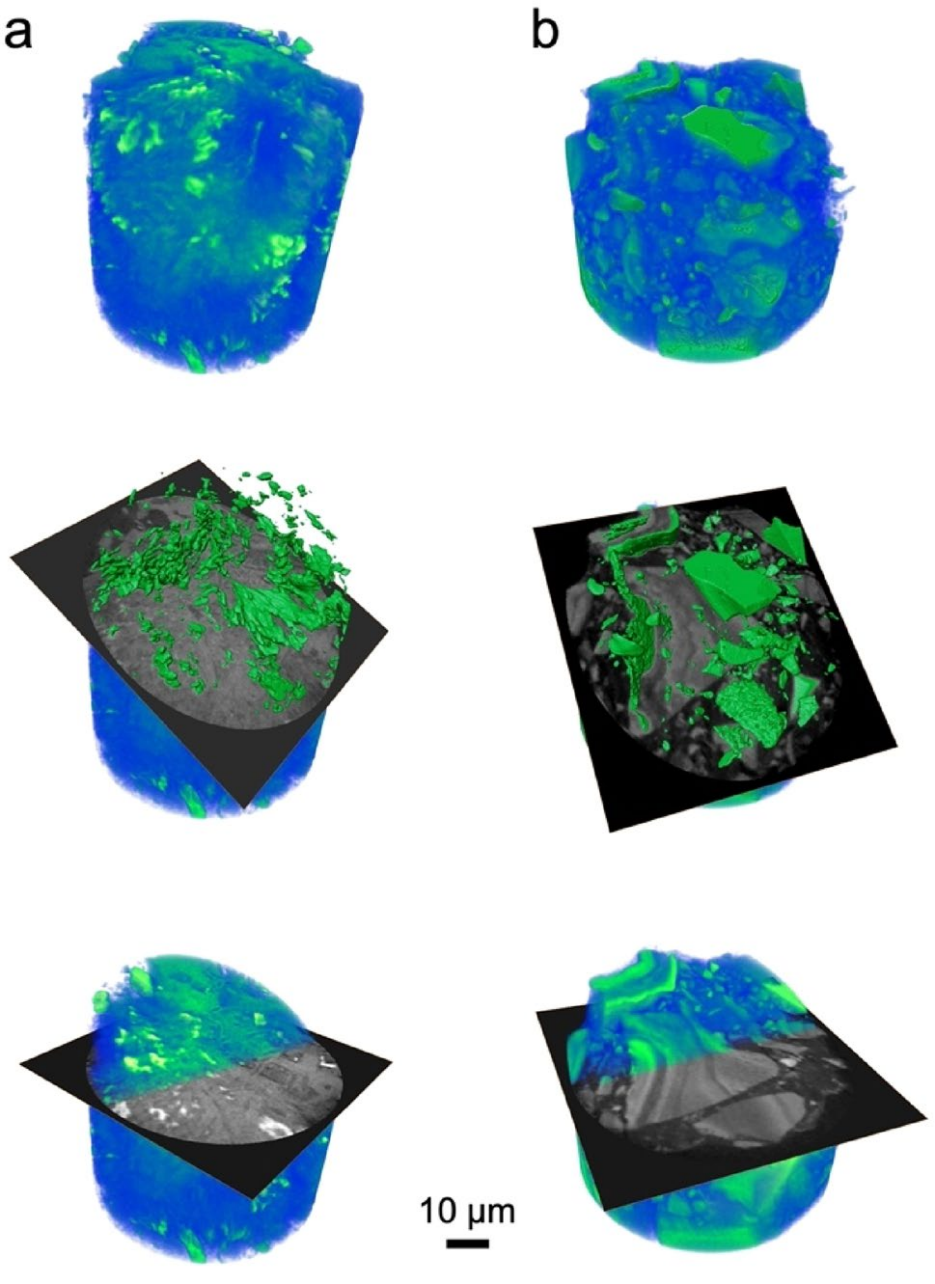
Nano-CT images of (**a**) GC sample P0-846 at 14 days of immersion in Tris buffer and (**b**) P0 glass under the same conditions, as discussed previously^15^. Volumetric reconstructions of the samples with a virtual cut (centre and bottom). The phase with the brightest contrast is highlighted in green.

To investigate the effect of crystallisation on microstructural changes during immersion, nano-CT images of P0-846 and P0 at 14 days of immersion were compared (Fig. 7). The areas with the brightest contrast (i.e. the highest electron densities) are marked in green. In our previous study investigating glass P0, those areas were interpreted as fluorite particles, embedded into the ion-depleted glass matrix^15^. A similar interpretation applies for immersed glass–ceramic P0-846: xonotlite, wollastonite and the amorphous matrix could not be discerned in the nano-CT image due to their similar density, whereas fluorite particles were distinct owing to their higher density. Fluorite particles in P0-846 occupied a smaller region than in P0 at 14 days of immersion. In addition, the particles’ morphology was different, as a smaller particle size with rounded edges and a lower degree of agglomeration was observed for the GC compared to the glass.

The absence of the large angular particles here, which were present in the glass sample, is explained by the increased degradation of the GC. By comparison, glass P0 was relatively inert, which prevented particle degradation and maintained their angular shape. Fluorite crystals precipitated on the particle surface, where they have grown to sizes of up to several tens of micrometres (Fig. 7b). The observed variation in surface structure probably originates from differences in nucleation and growth conditions in different areas of the glass particle surface^15^. By contrast, the multi-phase character of the GC has caused degradation along the interfaces, breaking the angular GC particles apart (Fig. 7a). The depletion in intergrown cuspidine and xonotlite in P0-846 created voids, which were well-suited for fluorite crystal nucleation but had a growth-limiting effect due to their size. Thus, at 14 days of immersion, the fluorite particles in P0-846 were smaller compared to those in P0. Additionally, P0-846 showed smaller numbers of fluorite particles compared to the parent glass, possibly because of the delaying effect of xonotlite on fluorite precipitation as discussed above.

Our results show that phosphate-containing GC show the capacity to induce apatite precipitation in Tris buffer solution. Ca release was comparable to the corresponding parent glasses, owing to a large amount of residual glassy matrix with an NC similar to that of the parent glass. GC also showed pronounced degradation of silicate crystal phases, leading to the formation of nano-sized channels and voids present in the material. Here, the GC containing high PSR showed almost complete degradation of amorphous and crystalline silicate networks within 14 days of immersion, since they contain only a minor amount of the relatively stable wollastonite. By contrast, GC with low PSR retained larger amounts of silicates, mainly ion-depleted silica, xonotlite and wollastonite.

An increase in PSR favoured apatite mineralisation (most likely a fluoride-substituted apatite) for glasses and GC, while smaller PSR favoured fluorite formation. FAp crystals present in the unreacted GC acted as nucleation sites, accelerating further apatite precipitation upon immersion. Additionally, xonotlite has a phosphate-binding effect in solution and, hence, potentially improves apatite formation in vitro. As a result of xonotlite crystallisation, excessive fluoride release appeared to be limited, resulting in an improved, i.e. lower, fluorite-to-apatite ratio for GC compared to the respective glasses.

## Conclusions

Taken together, the GC system studied here presents a promising starting point for further development of biodegradable implant materials. The system allows for customising the amounts of residual glassy matrix, FAp, wollastonite, xonotlite and cuspidine in the GC by adjusting the phosphate-content in the parent glass and the heat-treatment profile. This, in turn, allows for tailoring GC degradation. Together with tailoring the amount of FAp, this is considered beneficial, as it directly affects processes such as implant mineralisation and degradation. Our study also shows that the popular view that crystallisation of bioactive phospho-silicate glasses impedes degradation and, thus, apatite formation and bioactivity, needs to be reconsidered.

## Methods

### Glass design and synthesis

Glasses were based on the system SiO_2_–P_2_O_5_–CaO–CaF_2_ (Table 3). Glass P2 is a modification of the well-known Bioglass 45S5, altered by adding 9 mol% of CaF_2_ and completely replacing Na_2_O by CaO while maintaining the network connectivity (NC) at 2.11^15^. The PSR was varied by increasing the phosphate content together with stoichiometric amounts of calcium oxide, to maintain phosphate being present as orthophosphate in the glass. Our previous results showed that this maintained the structure of both silicate and phosphate phase^15^. A fixed amount of CaF_2_ was included in all compositions to allow crystallisation of FAp as well as to control the melting temperature. NC was calculated from the molar percentages of glass components according to the following equation:

**Table 3:** Nominal compositions (mol%) of the SiO_2_–P_2_O_5_–CaO–CaF_2_ glasses, together with the temperature of crystallisation treatment (T_P1_, in °C). A comparison of nominal and analysed fluoride contents is provided in Supplementary Table 1.

Glass	SiO_2_	P_2_O_5_	CaO	CaF_2_	T_P1_	GC
P0	46.8	0.0	44.2	9.0	846	P0-846
P1	44.3	1.2	45.5	9.0	894	P1-894
P2	41.9	2.4	46.7	9.0	901	P2-901
P3	39.9	3.4	47.7	9.0	884	P3-884
P4	38.0	4.3	48.7	9.0	816	P4-816
P5	36.3	5.1	49.6	9.0	778	P5-778

1 $${\text{NC}}= \frac{4 {{\text{SiO}}}_{2} - 2\mathrm{ CaO }+ 6 {{\text{P}}}_{2}{{\text{O}}}_{5}}{{{\text{SiO}}}_{2}}$$

Glasses were prepared in 150 g batches containing mixtures of analytical grades of the following raw materials: SiO_2_, Ca(H_2_PO_4_)_2_·2 H_2_O, CaCO_3_ and CaF_2_. The melting procedure is described elsewhere^15^. Part of the cast blocks was crushed to granules using a steel mortar and pestle. After sieving the glass granules to a particle size between 125 and 250 µm, the powdered samples were stored in a desiccator until use.

### Preparation of glass–ceramics

To obtain GC, glass samples were heat-treated at the temperature of their first crystallisation peak, T_P1_ (Table 3) in air. T_P1_ was obtained from differential scanning calorimetry experiments as described previously^29^. For this purpose, glass powder was given into platinum pans and placed inside a furnace (Nabertherm P330, Germany) for one hour after the target temperature was reached. GC sample names correspond to the code of the parent glass (i.e., the P_2_O_5_ content) and the temperature of heat-treatment, e.g. P0-846 (Table 3). Following the heat-treatment, the sintered bodies were air-quenched at room temperature. The GC were re-crushed with an agate mortar and pestle and sieved to a particle size between 125 and 250 µm. Further details on glass–ceramic preparation are provided elsewhere^29^.

### Immersion in Tris buffer solution

Immersion experiments were performed in 0.062 mol L^−1^ Tris–HCl buffer solution of an initial pH of about 7.3. A quantity of 50 mL was pipetted into a polyethylene container, the pH was determined using a pH electrode and meter (S40 SevenMulti™, Mettler Toledo) and 75 mg of glass or GC powder were added. The solutions were kept in an orbital shaker at 100 rpm and 37 °C. Experiments were performed in triplicate; Tris buffer solution without immersed samples was used as control. When reaching the time point of 6 h, 1, 2, 3, 7 or 14 days, the pH was measured and 1 mL of solution was removed for elemental concentration analysis. Afterwards the solution with the glass or GC particles was filtered through medium porosity filter paper. The remaining powder was rinsed with acetone to stop the reaction and left in air to dry.

Inductively coupled plasma optical emission spectroscopy (ICP-OES; 5110, Agilent Technologies, Germany) was used to analyse the concentrations of silicon, calcium and phosphorus in solution. Before the analysis, 1 ml of test solution was diluted with 9 mL of 1 mol L^−1^ concentrated nitric acid; calibration standards for Ca, Si and P were also prepared using 1 mol L^−1^ HNO_3_. The results are presented as a percentage of the respective ion originally present in the untreated glass (mean ± standard deviation of triplicate samples).

### Structural characterisation

Identification of crystalline phases was performed using X-ray diffraction (XRD; MiniFlex 300, Rigaku, Japan), with Cu-K_α_ radiation (30 kV; 10 mA). The diffraction patterns were recorded for a 2θ range from 5° to 75° with a step size of 0.02° at a scanning rate of 5°min^−1^. Full pattern Riedtveld refinement was accomplished using MAUD software with crystallographic information files of cuspidine (CIF 9,014,740), wollastonite (CIF 9,005,777), xonotlite (CIF 9,009,724), FAp (CIF 2,104,744) and fluorite (CIF 2,300,449) from crystallography open database (COD).

Additionally, attenuated total reflection Fourier transform infrared spectroscopy (ATR-FTIR; Cary 630, Agilent Technologies, USA) was carried out in absorbance mode. For this purpose, GC powders were finely ground with an agate mortar and pestle. Spectra were collected between 4000 and 400 cm^−1^ with a resolution of 4 cm^−1^ and 16 scans per measurement. Initial background measurements were recorded before each set of samples.

### Microstructural characterisation by (Scanning) Transmission Electron Microscopy ((S)TEM) and X-ray Microscopy (nano-CT)

For TEM characterisation, P0-846, P2-901 and P5-778 GC powders at 14 days of immersion in Tris buffer were dispersed in ethanol. A droplet of this suspension was dried on a carbon-coated copper grid and mounted in the TEM sample holder. TEM bright field images were recorded with an FEI Tecnai G2 F20 microscope (Thermo Fisher Scientific Company) operated at 200 kV, with a camera length of 200 mm. Energy dispersive X-ray spectroscopy (EDX) of Si (K-line), P (K-line), Ca (K-line) and F (K-line) were also recorded. Elemental quantification was performed by the software TIA (Thermo Fisher Scientific Company).

Additionally, powder P0-846 at 14 days of immersion was imaged using X-ray nano-computed tomography (nano-CT; Carl Zeiss Xradia 810 Ultra X-ray microscope) equipped with a chromium source (5.4 keV). For this purpose, a polyimide tube (Goodfellow Cambridge Ltd., LS522958) glued onto the tip of a metallic pin was filled with the powder and inserted into the sample holder.

The samples were scanned using Zernike phase contrast, a field-of-view of 64 µm^2^ with a detector binning of two (isotropic pixel size 128 nm^2^) and a total of 901 projection images. Projection images were collected for 180 degrees, with an exposure time of 20 s per projection. Sample drift was corrected using the adaptive motion compensation included in the device imaging software, which performs a scan with limited number of projections and the images obtained are used to correct large sample drifts. Image reconstruction was performed by a filtered back-projection algorithm using XMReconstructor software. The tomograms obtained were then exported as a stack of 16-bit TIFF images and visualised with Avizo (Thermo Fisher Scientific, version 9.4). Images were processed with median filter and segmentations were done visually, using the interactive threshold module.

### Supplementary Information

Supplementary Information.

## Data Availability

Data are available upon request to the corresponding authors.
