# Supplementary material for: Bioactive glass–ceramics containing fluorapatite, xonotlite, cuspidine and wollastonite form apatite faster than their corresponding glasses
Source: Sci Rep. 2024 Feb 18;14:3997. doi: 10.1038/s41598-024-54228-0 (PMC10874964; doi:10.1038/s41598-024-54228-0)
Supplement: Supplementary file 1 — Supplementary Information. [file 41598_2024_54228_MOESM1_ESM.docx]

Bioactive glass-ceramics containing fluorapatite, xonotlite, cuspidine and wollastonite form apatite faster than their corresponding glasses

Gloria Kirste^1,6^, Altair Contreras Jaimes^1^, Araceli de Pablos-Martín^2,1^, Juliana Martins de Souza e Silva^2^,^3^ Jonathan Massera^4^, Robert G. Hill^5^, Delia S. Brauer^1*^

^1^ Otto Schott Institute of Materials Research, Friedrich Schiller University, Fraunhoferstr. 6, 07743 Jena, Germany

^2^ Fraunhofer Institute for Microstructure of Materials and Systems IMWS, Walter-Hülse-Str. 1, 06120 Halle, Germany

^3^ Institute of Physics, Martin Luther University Halle-Wittenberg, Heinrich-Damerow-Str. 4, 06120 Halle, Germany

^4^ Faculty of Medicine and Health Technology, Tampere University, Korkeakoulunkatu 3, 33720 Tampere, Finland

^5^ Dental Physical Sciences, Barts and the London School of Medicine and Dentistry, Queen Mary University of London, Mile End Road, London E1 4NS, UK

^6^ Leibniz Institute for Solid State and Materials Research (IFW), Helmholtzstr. 20, 01069 Dresden, Germany

^#^ current address: Wilhelm Dyckerhoff Institut, Dyckerhoff GmbH, Dyckerhoffstraße 7, 65203 Wiesbaden, Germany

* Corresponding author: Prof. Delia S. Brauer, e-mail: [delia.brauer@uni-jena.de](mailto:delia.brauer@uni-jena.de), phone number: +49 3641-948-510

# Supplementary Material

(For references, please refer to the main manuscript.)

*Effect of fluoride loss on network connectivity (NC)*

The glasses’ source of fluoride, CaF_2_, does not directly affect the glasses' NC (Eq. 1, see main paper), since fluoride ions are known to complex Ca^2+^ ions with no Si-F bonds being formed^15,52,53^. However, fluoride loss upon glass melting can cause a change in NC if fluoride loss reactions involve any of the glass components SiO_2_, CaO or P_2_O_5_. As shown in our recent publication on fluoride loss in the present glass system, the dominating mechanisms of fluoride loss here are the formation and evaporation of the volatile compounds HF and POF_3_. ^33^

HF volatilisation occurs throughout the entire glass series *via* reaction of CaF_2_ with water, originating from raw materials or atmospheric humidity (Eq. 2). As a consequence, the amount of network-modifying CaO increases and causes NC to decrease (Eq. 1).

$\mathrm{CaF}_{2} + H_{2}O \to2 HF\uparrow+ CaO$ (2)

Phosphate-containing glasses may also undergo an additional fluoride-loss mechanism *via* POF_3_ formation involving the consumption of P_2_O_5_ (Eq. 3), a fluoride-loss route which increases with increasing P_2_O_5_ content in the glass^33^. This reaction affects NC in two ways: by reducing the amount of P_2_O_5_ in the glass and by forming the modifier CaO. The reaction shown in Eq. 3 has, thus, twice the impact on NC decrease as the one of the reaction shown in Eq. 2 (since the effect of P_2_O_5_ on NC is three times larger than that of CaO, Eq. 1, and P_2_O_5_ and CaO are formed at a stoichiometric ratio of 1:3, Eq. 3).

$3 \mathrm{CaF}_{2}+P_{2}O_{5} \to2 \mathrm{POF}_{3}\uparrow+ 3 CaO$ (3)

For the calculation of the results shown in Fig. 1, the combined fluoride loss mechanisms *via* HF and POF_3_ formation were taken into account. The extent of fluoride loss *via* HF was estimated based on the one observed for glass P0, since this composition can only lose fluoride *via* the HF route. Additional fluoride losses in the phosphate-containing glasses were therefore assigned to losses *via* POF_3_ formation. We therefore did not take into account the possibility of increased fluoride loss *via* HF formation caused by reactions with water originating from Ca(H_2_PO_4_)_2_·2 H_2_O used as the phosphate source.

*Calculation of the composition of the residual glassy matrix*

Since the relative amount of residual glassy (amorphous) matrix in the GC was not accessible *via* full pattern refinement, we estimated the residual glass compositions as a function of the unknown final amount of glassy matrix $x$ in the analysed samples. Hence, the curves in Fig. 1 do not represent a progressive change in residual glass composition with time during the crystallisation process, as the crystallisation sequence was not considered here. Instead, it shows the expected glass composition at one hour of heat treatment assuming that 20 to 90 at% of the total sample composition are present as crystalline phases (ratios given in Table 2).

The residual glass composition, $\chi_{i, res}$, was calculated by comparing the parent glass composition (after fluoride loss), $\chi_{i, par}$, with the chemical composition of the GC’s crystalline part, $\chi_{i, cryst}$. Here, the index $i$ represents the involved elements Si, Ca, P, O and F. First, the weight fractions of the crystalline phases, obtained from pattern refinement in MAUD, were converted to the atomic composition of the cumulated crystalline parts for each GC (before immersion). For simplicity's sake, this conversion was accomplished using the molar masses of stoichiometric FAp (Ca_5_(PO_4_)_3_F), cuspidine (Ca_4_Si_2_O_7_F_2_), xonotlite (Ca_6_Si_6_O_17_F_2_) and wollastonite (CaSiO_3_) without any substitutions. Next, the nominal glass compositions from Table 1 were corrected for P_2_O_5_ and CaF_2_ loss during melting as described above before being converted to atomic percentages as well.

Subsequently, we reconstructed the atomic composition of the residual glassy matrix according to the following equation:

$\chi_{i, par}= x\cdot\chi_{i, res}+\left( 1-x \right)\cdot\chi_{i, cryst}$ (5)

Finally, $\chi_{i, res}$ was converted back to the molar percentages of the glass components SiO_2_, CaO, CaF_2_ and P_2_O_5_ and plotted as a function of $x$.


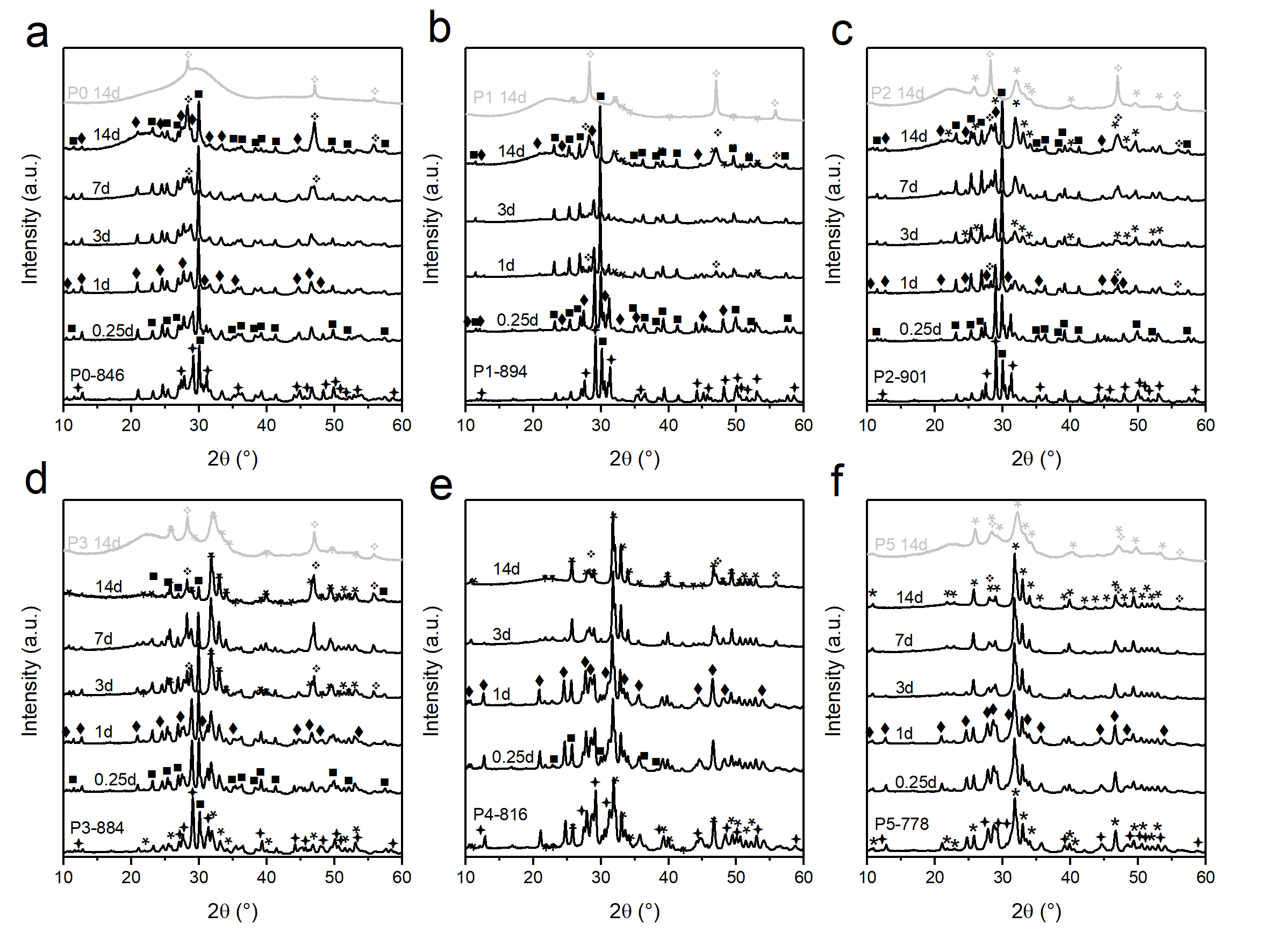


**Supplementary Figure 1**: X-ray diffraction patterns at different time points of immersion for GC a) P0-846, b) P1-894, c) P2-901, d) P3-884, e) P4-816 and f) P5-778, labelling of reflection peaks:
**🟄** cuspidine, **▼** wollastonite, **⧫**xonotlite, 🞹 apatite, ❖ fluorite


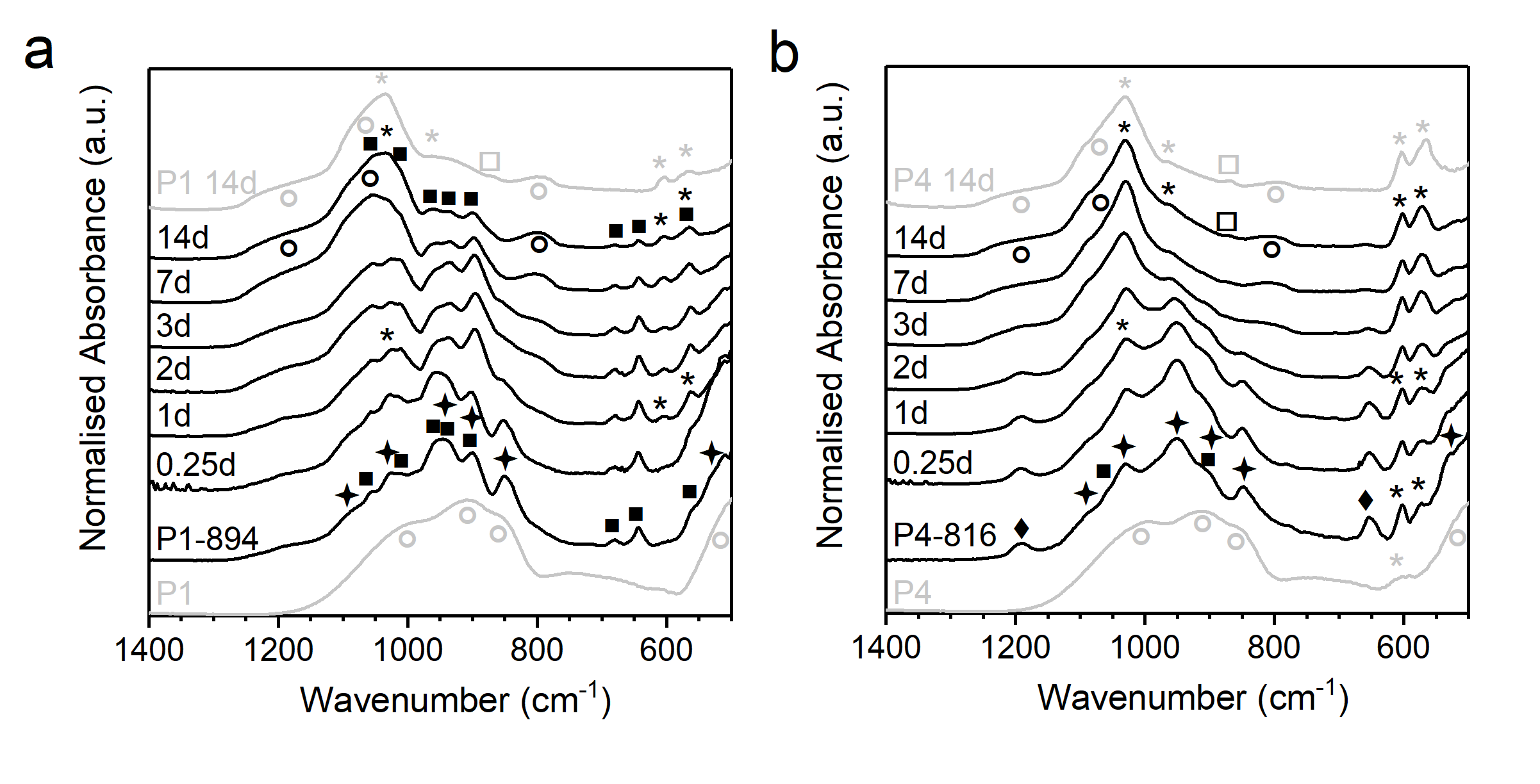


**Supplementary Figure 2**: FTIR spectra at different time points of immersion for a) P1 and P1-894 and b) P4 and P4-816. Parent glasses for comparison plotted in grey, labelling of vibration bands: **⭘** amorphous Si-O,**🟄** cuspidine, **▼** wollastonite, **⧫** xonotlite, 🞹 apatite/phosphate, △ carbonate.


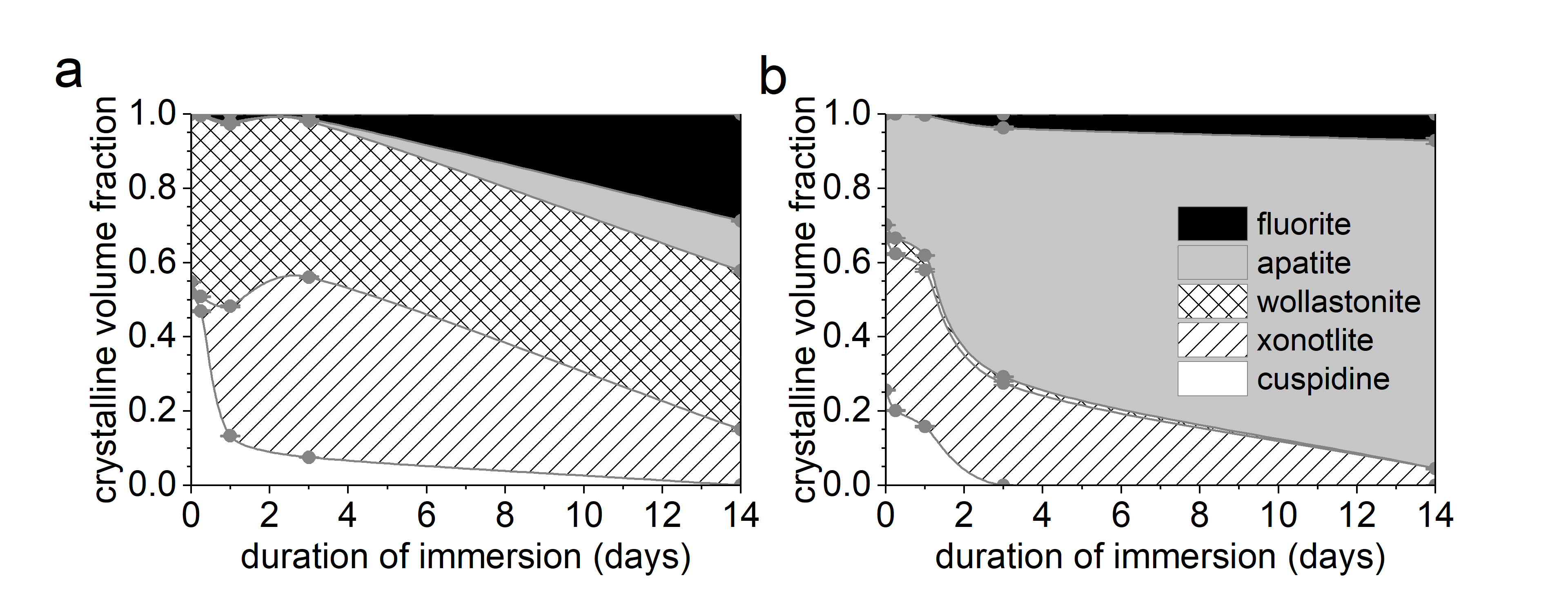


**Supplementary Figure 3**: Volume fractions of cuspidine, xonotlite, wollastonite, apatite and fluorite within the crystalline share of the GC at different time points of immersion, calculated by full pattern Rietveld refinement for a) P1-894 and b) P4-816 (Lines are drawn as a visual guide only; original diffraction patterns are depicted in Supplementary Figure 1.)

**Supplementary Table 1**: Nominal and analysed^16^ fluoride concentrations (± standard deviation, SD) in the glass samples.

| **Glass** | **Nominal F (wt.%)** | **Analysed F (wt.%) ± SD** |
| --- | --- | --- |
| P0 | 5.70 | 4.96 ± 1.02 |
| P2 | 5.53 | 3.20 ± 1.43 |
| P3 | 5.46 | 4.02 ± 1.16 |
| P5 | 5.35 | 2.42 ± 1.07 |
